# Major ocular trauma in Taiwan: 2002–2004 versus 2012–2014

**DOI:** 10.1038/s41598-018-25030-6

**Published:** 2018-05-04

**Authors:** Yi-Sheng Chang, Yu-Ti Teng, Yi-Hsun Huang, Mei-Ling Liu, Jia-Horung Hung, Sheng-Min Hsu, Fu-Chin Huang, Min-Hsiu Shih, Wan-Ju Chen, Chun-Chieh Lai, Shu-Fang Hsiao, Shih-Hao Wang, Sung-Huei Tseng

**Affiliations:** 1 Department of Ophthalmology, College of Medicine, National Cheng Kung University, Tainan, Taiwan; 2 Department of Ophthalmology, National Cheng Kung University Hospital, College of Medicine, National Cheng Kung University, Tainan, Taiwan; 3 Institute of Clinical Medicine, College of Medicine, National Cheng Kung University, Tainan, Taiwan

## Abstract

We investigated the temporal changes in major eye injuries in Taiwan by reviewing the medical records of all patients with ocular trauma hospitalized at the National Cheng Kung University Hospital during 2002–2004 and 2012–2014. A total of 169 eyes (161 patients) during 2002–2004 and 121 eyes (120 patients) during 2012–2014 were enrolled (mean ± SD age: 41.9 ± 20.8 years in 2002–2004, and 51.8 ± 19.3 years in 2012–2014). Males accounted for ~75% of patients. The most frequent injury-causing object was metallic material (~24%), and blunt traumas were most frequently attributable to traffic accidents and falls. The most common locations of injuries for males and females were the workplace and home, respectively. Open-globe injuries occurred in ~70% of eyes, requiring primary repair, cataract extraction, and/or intraocular lens implantation. The frequencies of fall injury, lacrimal system laceration, lens injury, corneal/scleral foreign bodies, and use of intracameral antibiotics increased from 2002–2004 to 2012–2014, while those of closed-globe injury, vitreous haemorrhage, optic nerve injury, and medical treatment decreased. The final visual acuity remained poor (≤20/200) in >1/3 of injured eyes. Despite therapeutic advancements, major eye injuries still pose a high risk for poor visual outcome.

## Introduction

Eye injuries are the leading cause of monocular blindness. Annually, more than 500,000 blinding injuries occur worldwide, and even more injuries cause partial loss of sight^1^. Moreover, major ocular trauma causes individual suffering and loss of productivity, and carries societal costs related with the increased use of medical care and rehabilitation services.

Several population- and nation-based studies have estimated the annual incidence rate of hospitalized ocular trauma as a principal diagnosis in 8.14–13.3/100,000 population^2–6^. However, these studies were limited by the unavailability of original medical records to delineate clinical profiles such as the circumstances of ocular trauma, mechanism and severity of eye injuries, specific treatment, sequelae, and visual outcome, which can only be identified through hospital-based studies. Accordingly, the rationale for performing this hospital-based study is that the epidemiology of ocular trauma is time- and region-dependent and there are limited hospital-based studies published on this topic^7–10^, particularly from Asia^11–15^. To our knowledge, studies investigating the decade changes of the clinical characteristics of major ocular trauma patients requiring hospitalization are particularly scarce^16,17^. We hypothesized that the epidemiology of ocular trauma has changed in accordance with the modernization of society, and therefore conducted this study on the profiles of hospitalized major ocular trauma cases in 2002–2004 versus 2012–2014 in order to add to the body of knowledge regarding the epidemiology and clinical features of ocular injury. We further compared our results to those of studies conducted in western and other Asian populations.

## Patients and Methods

This retrospective study was performed in accordance with the Declaration of Helsinki and relevant guidelines and regulations, and was approved by the Institutional Review Board of the National Cheng Kung University Hospital (NCKUH; approval code A-ER-104–207), which waived the need for informed consent because patient anonymity was maintained by the data source. The NCKUH is one of two tertiary referral centres in the Tainan City (estimated population, 1.85–1.88 million during 2002–2014) in southern Taiwan and provides a 24-hour emergency service for the general public in the city and surrounding small towns. We reviewed the medical records of all hospitalized patients in the Department of Ophthalmology who presented with ocular trauma to the Emergency Department of our hospital from 1 January, 2002 to 31 December, 2004 and from 1 January, 2012, to 31 December, 2014. We recorded and analysed patient age and sex; month of year, day of the week, time of day, mechanism of injury, and other information regarding the circumstances of the ocular trauma; ophthalmologic findings; treatments provided; sequelae; and final visual acuity.

Based on the presence of a full-thickness wound on the eye wall (cornea and sclera) or not, eyeball trauma was classified as an open- or closed-globe injury^18^. Open-globe injuries included eyeball rupture (full-thickness wound of the eye wall caused by a blunt trauma), penetrating injury (single laceration of the eye wall usually caused by a sharp object), perforating injury (two full-thickness lacerations with an entrance and an exit), and intraocular foreign body. Closed-globe injuries included contusion, corneal/scleral lamellar laceration, and thermal or chemical burn. Injuries outside the eyeball were grouped as adnexal injuries such as lacrimal system laceration, optic nerve injury, extra-ocular nerve injury, or blowout fracture.

Categorical variables were presented as number and percentage values and were analysed using the chi-squared test. Continuous variables were presented as mean, standard deviation (SD), and range, and were analysed using the Student’s *t* -test. All statistical analyses were performed using SPSS software, version 20 (IBM, Armonk, New York, USA).

## Results

### Demographics

**Figure 1:**
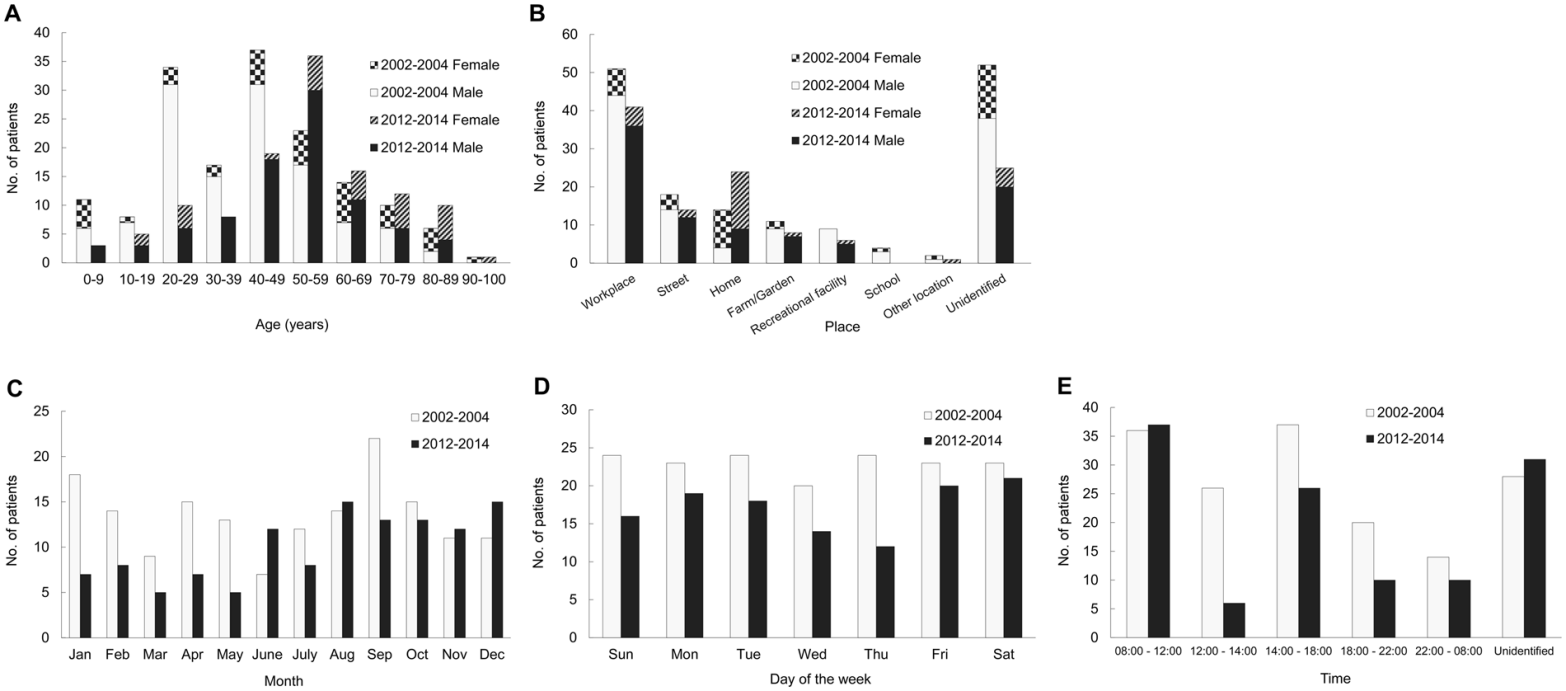
Distribution of patients admitted to a university hospital in Taiwan with a primary diagnosis of ocular trauma in 2002–2004 (n = 161) versus 2012–2014 (n = 120). (**A**) Age and sex of the patients. (**B**) Place of injury. (**C**) Month of the year. (**D**) Day of the week. (**E**) Time of day.

In 2002–2004, 161 patients were admitted to our hospital for treatment of 169 injured eyes, while in 2012–2014, 120 patients were admitted with 121 injured eyes. As shown in Table 1, in both study periods, males accounted for approximately thrice as many injuries, and were about 10 years younger than females (*P* = 0.01 in 2002–2004, and *P* = 0.03 in 2012–2014). As seen in Fig. 1A and Table 1, the peak ages in 2002–2004 were 20–29 and 40–49 (mean ± SD: 41.9 ± 20.8) years, which were significantly younger than the peak age of 50–59 (mean ± SD: 51.8 ± 19.3) years in 2012–2014. The laterality of the eye injury did not significantly differ between the time periods.

**Table 1:** Demographics of 161 patients (169 eyes) in 2002–2004 versus 120 patients (121 eyes) in 2012–2014 hospitalized for ocular trauma.

Characteristics	No. of patients (%)		*P*-value
	2002–2004	2012–2014	
Age, years, mean ± SD (range)	41.9 ± 20.8 (1.8–91)	51.8 ± 19.3 (2–91)	<0.001^a^
Male	39.1 ± 18.1 (4–86)	49.2 ± 17.1 (2–83)	<0.001^a^
Female	50.6 ± 25.7 (1.8–91)	59.5 ± 22.8 (13–91)	0.13
Sex			0.76
Male	122 (75.8)	89 (74.2)	
Female	39 (24.2)	31 (25.8)	
Eye injured			0.13
Right	74 (46.0)	54 (45.0)	0.87
Left	79 (49.1)	65 (54.2)	0.40
Both	8 (5.0)	1 (0.8)	0.05
Source of referral			0.09
Directly visiting our Emergency Department	68 (42.2)	63 (52.5)	0.13
Local practitioners	70 (43.5)	46 (38.3)	0.39
Local hospitals	20 (12.4)	7 (5.8)	0.06
Other medical centres	3 (1.9)	4 (3.3)	0.43
Follow-up, mean ± SD, months (range)	5.8 ± 7.2 (2 d–36 mo)	6.6 ± 4.7 (2 w–23 mo)	0.25

^a^*P* < 0.05.

Most patients with ocular trauma either directly visited our Emergency Department or were referred from local practitioners. The mean follow-up duration was around 6 months during both time periods.

### Location, timing, and objects/activities causing ocular trauma

Figure 1B shows that the most common location of ocular trauma for male patients in both study periods was the workplace, followed by the street. However, female patients were most commonly injured at home, followed by the workplace. Injuries at home were more common in the 2012–2014 period.

As shown in Fig. 1C, injuries were more common in January and September during 2002–2004, and August and December during 2012–2014. There was no significant difference in the incidence of trauma by day of the week in 2002–2004, whereas trauma was sustained most frequently on Friday and Saturday during 2012–2014 (Fig. 1D). Injuries occurred most frequently during working hours (from 8 a.m. to 12 p.m. and from 2 to 6 p.m.) and less commonly during sleeping hours (from 10 p.m. to 8 a.m.) during both time periods (Fig. 1E).

Table 2 shows the frequency with which various objects or activities caused ocular trauma among the patients in our study. Pieces of metal remained the most common cause of injury (23.6% in 2002–2004 and 24.2% in 2012–2014). The frequency of falling trauma significantly increased and injury by fingernail(s) significantly decreased from 2002–2004 to 2012–2014.

**Table 2:** Objects/activities causing ocular trauma in the 161 patients (169 eyes) hospitalized in 2002–2004 versus the 120 patients (121 eyes) hospitalized in 2012–2014.

Objects/activities	No. of patients (%)		*P*-value
	2002–2004	2012–2014	
Sharp object injury			
Metal material	38 (23.6)	29 (24.2)	0.91
Other sharp object	16 (9.9)	13 (10.8)	0.81
Fingernail(s)	12 (7.5)	0 (0.0)	0.002^a^
Knife	2 (1.2)	3 (2.5)	0.43
Mixed or blunt object injury			
Unspecified blunt object	16 (9.9)	18 (15.0)	0.20
Traffic accident	12 (7.5)	11 (9.2)	0.60
Fall	12 (7.5)	18 (15.0)	0.04^a^
Plant material/branch	7 (4.3)	4 (3.3)	0.66
Lawn mowing	6 (3.7)	7 (5.8)	0.41
Ball	6 (3.7)	3 (2.5)	0.56
Stone	5 (3.1)	1 (0.8)	0.19
Hand/fist	4 (2.5)	3 (2.5)	0.99
Toy gun	1 (0.6)	0 (0.0)	0.39
Burn injury			
Chemical burn	4 (2.5)	2 (1.7)	0.64
Fireworks	3 (1.9)	0 (0.0)	0.13
Thermal burn	1 (0.6)	1 (0.8)	0.83
Others	5 (3.1)	4 (3.3)	0.92
Unidentified	11 (6.8)	3 (2.5)	0.10

^*a*^*P* < 0.05.

### Mechanisms of injuries

As summarized in Table 3, which lists the mechanisms of injuries, around 70% of the eyes in both periods suffered from open-globe injuries, which remained much more prevalent than closed-globe or adnexal injuries. Similarly, eyeball rupture and penetrating injury each accounted for around 30% of the eye injuries in both periods, whereas the number of injuries caused by an intraocular foreign body decreased in the 2012–2014 period; there were no cases of perforating injury. Closed-globe injuries requiring hospitalization remarkably decreased in both number and percentage in the 2012–2014 period. Around 10% of patients in both periods had adnexal injuries, some of whom (16 eyes, 9.5%, in 2002–2004; and 2 eyes, 1.5%, in 2012–2014) had co-existing open- or closed-globe injuries.

**Table 3:** Mechanisms of injuries in the 169 eyes treated in 2002–2004 versus the 121 eyes treated in 2012–2014.

Mechanisms of injuries	No. of eyes (%)^b^		*P*-value
	2002–2004	2012–2014	
Open-globe injury	115 (68.0)	89 (73.6)	0.31
Eyeball rupture	52 (30.8)	40 (33.1)	0.68
Penetrating injury	47 (27.8)	43 (35.5)	0.16
Perforating injury	0 (0)	0 (0)	1.00
Intraocular foreign body	16 (9.5)	6 (5.0)	0.15
Closed-globe injury	51 (30.2)	22 (18.2)	0.02^a^
Adnexal injury	19 (11.2)	12 (9.9)	0.72

^a^*P* < 0.05.

^b^Total is greater than 100% because of co-existent injuries.

As seen in Table 4 listing injury types, the most common injuries were corneal/scleral laceration or limbal wound dehiscence (133 eyes, 79.4%, in 2002–2004 versus 79 eyes, 65.3%, in 2012–2014), hyphaemia (69 eyes, 40.2% versus 39 eyes, 32.2%), and lens injury (39 eyes, 23.1% versus 41 eyes, 33.9%). In the 2012–2014 period, a significant increase was observed in lacerations of the lacrimal system, lens injuries (particularly lens subluxation), and corneal/scleral foreign bodies, whereas significant decreases were observed in vitreous haemorrhage and optic nerve injuries compared to the 2002–2004 period.

**Table 4:** Injuries and their complications in the 169 eyes treated in 2002–2004 versus the 121 eyes treated in 2012–2014.

Injuries/complications	No. of eyes (%)		*P*-value
	2002–2004	2012–2014	
Laceration (partial/full thickness)	137 (81.1)	100 (82.6)	0.73
Cornea	72 (42.6)	48 (39.7)	0.62
Sclera	18 (10.7)	13 (10.7)	0.98
Corneoscleral	22 (13.0)	9 (7.4)	0.13
Limbal wound dehiscence	21 (12.4)	19 (15.7)	0.43
Lacrimal system	3 (1.8)	11 (9.1)	0.004^a^
Extraocular muscles	1 (0.6)	0 (0.0)	0.40
Burn	8 (4.7)	3 (2.5)	0.32
Thermal	2 (1.2)	1 (0.8)	0.77
Acidic	3 (1.8)	0 (0.0)	0.14
Alkali	3 (1.8)	2 (1.7)	0.94
Corneal ulceration	11 (6.5)	5 (4.1)	0.38
Hyphaemia	69 (40.2)	39 (32.2)	0.14
Iridodialysis	9 (5.3)	9 (7.4)	0.46
Secondary glaucoma	15 (8.9)	7 (5.8)	0.33
Lens injury	39 (23.1)	41 (33.9)	0.04^a^
Traumatic cataract	22 (13.2)	26 (21.5)	0.06
Lens subluxation	3 (1.8)	8 (6.6)	0.03^a^
Lens dislocation	9 (5.3)	3 (2.5)	0.23
Intraocular lens dislocation	5 (3.0)	4 (3.3)	0.87
Vitreous haemorrhage	29 (17.2)	11 (9.1)	0.05^a^
Retinal injury	23 (13.6)	13 (10.7)	0.47
Macular hole	2 (1.2)	0 (0.0)	0.23
Retinal oedema	6 (3.6)	2 (1.7)	0.33
Retinal haemorrhage	6 (3.6)	1 (0.8)	0.14
Retinal defect/break	4 (2.4)	4 (3.3)	0.63
Retinal detachment	5 (3.0)	6 (5.0)	0.38
Choroidal detachment	2 (1.2)	4 (3.3)	0.21
Endophthalmitis	4 (2.4)	0 (0.0)	0.09
Foreign bodies	22 (13.0)	20 (16.5)	0.40
Corneal/scleral	4 (2.4)	10 (8.3)	0.02^a^
Intraocular	16 (9.5)	6 (5.0)	0.15
Orbital	2 (1.2)	4 (3.3)	0.21
Optic nerve injury	12 (7.1)	1 (0.8)	0.01^a^
Oculomotor nerve palsy	1 (0.6)	1 (0.8)	0.81
Orbital facture	3 (1.8)	0 (0.0)	0.14

^a^*P* < 0.05.

### Treatment modalities

Table 5 shows the modalities used to treat the injured eyes. The most frequent treatments were primary repair of corneal/scleral wounds, irrigation of the anterior chamber to remove blood or exudate, removal of a damaged lens (mostly combined with implantation of an intraocular lens), and vitrectomy. Of note, the use of intraocular antibiotics, particularly intracameral injection of cefuroxime to prevent or treat intraocular infection, increased significantly in the 2012–2014 period compared to that in the 2002–2004 period. Other increasingly used treatments in the 2012–2014 period included repair of the lacrimal system, pars plana lensectomy, and removal of corneal/scleral foreign bodies.

**Table 5:** Modalities of treatment in the 169 eyes in 2002–2004 versus the 121 eyes in 2012–2014.

Treatment	No. of eyes (%)		*P*-value
	2002–2004	2012–2014	
**Surgical procedures**			
Primary repair	113 (66.9)	94 (77.7)	0.04^a^
Corneal wound	54 (32.0)	44 (36.4)	0.43
Scleral wound	15 (8.9)	13 (10.7)	0.60
Corneoscleral wound	21 (12.4)	10 (8.3)	0.26
Dehiscent wound	19 (11.2)	16 (13.2)	0.61
Lacrimal system	3 (1.8)	11 (9.1)	0.004^a^
Extraocular muscle	1 (0.6)	0 (0.0)	0.40
Exploration of globe	2 ((1.2)	0 (0.0)	0.23
Irrigation of the anterior chamber	40 (23.7)	20 (16.5)	0.14
Iridoplasty	4 (2.4)	2 (1.7)	0.67
Removal of the lens	31 (18.3)	30 (24.8)	0.18
Aspiration	4 (2.4)	3 (2.5)	0.95
Extracapsular cataract extraction	7 (4.1)	6 (5.0)	0.74
Phacoemulsification	18 (10.7)	13 (10.7)	0.98
Pars plana lensectomy	2 (1.2)	8 (6.6)	0.01^a^
Intraocular lens implantation	30 (17.8)	18 (14.9)	0.52
In the bag	11 (6.5)	11 (9.1)	0.41
In the sulcus	5 (3.0)	2 (1.7)	0.48
Suture of intraocular lens	4 (2.4)	5 (4.1)	0.39
Vitrectomy	33 (19.5)	32 (26.4)	0.16
Anterior vitrectomy	21 (12.4)	18 (14.9)	0.55
Posterior vitrectomy	12 (7.1)	14 (11.6)	0.19
Treatment/prophylaxis of retinal detachment	21 (12.4)	19 (15.7)	0.43
Pneumatic retinopexy	2 (1.2)	3 (2.5)	0.40
Scleral buckle	8 (4.7)	2 (1.7)	0.16
Cryopexy	2 (1.2)	3 (2.5)	0.40
Laser	8 (4.7)	11 (9.1)	0.14
Silicone oil	1 (0.6)	0 (0.0)	0.40
Intraocular antibiotics	46 (27.2)	69 (57.0)	<0.001^a^
Intracameral injection	16 (9.5)	38 (31.4)	<0.001^a^
Intravitreal injection	30 (17.8)	31 (25.6)	0.11
Removal of foreign bodies	22 (13.0)	19 (15.7)	0.52
Corneal/scleral foreign bodies	4 (2.4)	10 (8.3)	0.02^a^
Intraocular foreign bodies	16 (9.5)	6 (5.0)	0.15
Orbital foreign bodies	2 (1.2)	4 (3.3)	0.21
Repair of orbital fracture	3 (1.8)	0 (0.0)	0.14
Evisceration	3 (1.8)	5 (4.1)	0.23
**Medical treatment only**	36 (21.3)	9 (7.4)	0.001^a^

^a^*P* < 0.05.

A second or third operative procedure was performed in 26 (15.4%) and 22 (18.2%) of the eyes in 2002–2004 and 2012–2014, respectively, to manage sequelae or complications such as traumatic/surgical aphakia, corneal scar, bullous keratopathy, or retinal detachment.

The number of hospitalized patients requiring medical treatment only without surgery significantly decreased from 2002–2004 (36 eyes, 21.3%) to 2012–2014 (9 eyes, 7.4%). These included eyes with corneal burns or trauma-related ulcerations, hyphaemia that resolved spontaneously, and optic neuropathy.

### Final visual acuity

**Figure 2:**
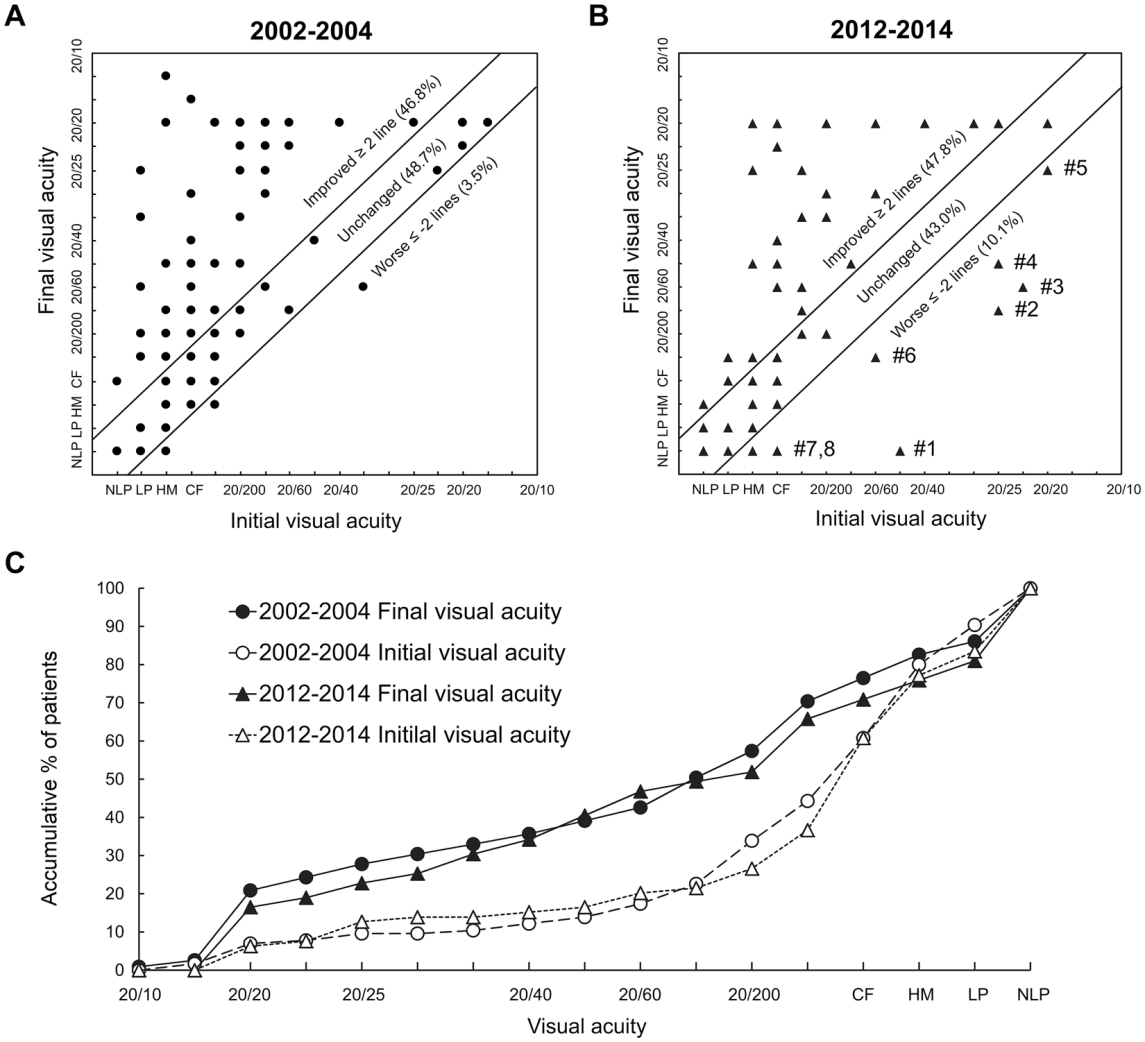
Initial versus final visual acuities in 115 injured eyes in 2002–2004 (**A**) and 79 injured eyes in 2012–2014 (**B**) with measurable visual acuities before and after treatment. (**C**) Accumulative percentages of patients by visual acuity. (**B**) Patient 1 had lens subluxation and secondary glaucoma but later developed retinal detachment requiring pars plana vitrectomy and silicone oil tamponade. Patients 2–5 had corneal penetrating injuries and later developed corneal scars. Patient 6 had lens subluxation and secondary glaucoma and had the sequela of aphakia. Patient 7 had severe scleral penetrating injury and Patient 8 had initial retinal detachment and traumatic cataract. Abbreviations: NLP, no light perception; LP, light perception; HM, hand movement; CF, counting fingers.

Figure 2 shows the initial versus final visual acuities in 115 eyes (68.0%) in 2002–2004 and 79 eyes (65.8%) in 2012–2014, in which the visual acuity could be measured before and after treatment. The visual acuity improved ≥2 lines after treatment in 55 (47.8%) and 37 (46.8%) eyes in 2002–2004 and 2012–2014, respectively.

Table 6 shows the comparison of final visual acuity between the two periods. In both periods, ~20% of eyes achieved a good visual outcome, ~20% had a moderate visual acuity, and over one-third had poor visual acuity.

**Table 6:** Final visual acuity in the 169 eyes treated in 2002–2004 versus the 121 eyes treated in 2012–2014.

Final visual acuity	No. of eyes (%)		*P*-value
	2002–2004	2012–2014	
Good (20/25 or better)	41 (24.3)	24 (19.8)	0.37
Moderate (20/100 to 20/28)	33 (19.5)	27 (22.3)	0.56
Poor (20/200 or worse)	62 (36.7)	41 (33.9)	0.62
Undocumented	33 (19.5)	29 (24.0)	0.36

## Discussion

Eye injury is an important public health concern worldwide because it causes significant morbidity that is often preventable. Our study, the first to report temporal changes in major ocular trauma in a Taiwanese population, showed that most patients admitted to our university hospital with a primary diagnosis of ocular trauma were young and middle-aged men and that most had an open-globe injury due to metal material at work. An important change over time was that the mean age of the patients increased by almost 10 years from 2002–2004 to 2012–2014. Moreover, fall injury, lacrimal system laceration, lens injury (particularly lens subluxation), corneal/scleral foreign bodies, and use of intracameral antibiotics during surgery increased in frequency from the first time period to the second. In contrast, features of major ocular traumas requiring hospitalization that decreased in frequency were closed-globe injury, vitreous haemorrhage, optic nerve injury, and medical treatment only. However, despite improvements in treatment, final visual acuity remained poor in more than a third of our cases in both study periods. From this comparison study, we delineated that the risks for major ocular trauma and vision prognosis after injury depend on a variety of factors.

A preponderance of male patients among those with eye injuries is a universal finding, with reported male:female ratios of 2.8:1 to 5.2:1 in Asia^11–15^, and even 5.5:1 to 7.8:1 in Italy^8,17^. In a study analysing patients presenting with ocular trauma to the Emergency Department at a medical centre in northern Taiwan, the overall male:female ratio for “all severities” of ocular trauma was 2.4:1^19^. By contrast, in this study focusing on “major” ocular trauma requiring hospitalization, the higher ratios of 3.1:1 in 2002–2004 and 2.9:1 in 2012–2014 reflect a higher risk of males with potentially vision-threatening eye injuries; these results are supported by a national cohort study conducted in Taiwan showing a ratio of 3.1:1^6^. Of note, the risk of injury by sex differed with age; for male patients in this study, the risk peaked for those aged 20–59 years, in which the male:female ratios were as high as 5.5:1 in 2002–2004 and 5.6:1 in 2012–2014. However, the male:female ratios were approximately equal for those aged 60 years or older (0.9:1 in 2002–2004, and 1.2:1 in 2012–2014).

The patient age in this study peaked at young and middle-aged adults, which correlates with results reported in studies of Italian^8^, Iranian^15^, Chinese^12,13^, and Taiwanese populations^11^. However, studies conducted in American^2,3^, Scottish^16^, Australian^10^, and Singaporean populations^5^ further showed a second peak in old age (80 years and older). The mean age of our patients was 41.9 years in 2002–2004 versus 51.8 years in 2012–2014. Comparing the two periods in this study, major ocular trauma in the 2012–2014 period decreased greatly in patients aged 20–49 years but increased in those aged 50–59 years.

For the young-adult and middle-aged males in our study, activity risk factors for major ocular trauma were metal-working, driving, social behaviours such as fighting, and participation in dangerous sports or hobbies, similar to findings from previous reports^7,8,11–14,17,19^. In contrast, women in our study were most often injured at home and the workplace. A universal finding in studies of work-related eye injuries is that 55% to 91% of patients were not using protective eye wear at the time of injury^20–23^. Of note, traffic accidents have also been determined as an important risk factor for ocular trauma throughout studies in Taiwan, accounting for 7.5–9.2% of patients in our study, 10.8% in Tsai *et al*.’s study^19^, and 20.4% in Lee *et al*.’s study^6^. Most patients involved in traffic accidents are young adolescents riding motorcycles, a common occurrence in Taiwan^24^.

A major finding in this study is that the mean age of patients in 2012–2014 was 10 years older than that in 2002–2004; the reason for this change could involve two explanations. First, over the past decade, young people in Taiwan have tended to engage in the business, service, high tech, electronics industries, academic research, and other low-risk work. By contrast, middle-age laborers engaging in high-risk work, such as construction, manufacturing, or agriculture, have elongated their working ages and are now retiring later than before^25^, thereby continuing to be exposed to high risks of ocular trauma over time. Second, a law implemented in 1997 enforcing motorcyclists to wear helmets has reduced the risk and severity of head and/or eye injuries decade by decade, particularly among young males^26,27^. The risk of ocular trauma related to working with hazardous materials or involvement in a traffic accident can be greatly reduced by such measures as using safety (laminated) glass in windscreens; wearing helmets, safety goggles, seat belts, and other protective gear appropriate to the high-risk activity^23^; and avoiding risky behaviours, such as drinking alcohol before driving or not following safety procedures for operating machinery. Education regarding these safety measures is essential to reduce the incidence and severity of ocular injuries. Evidence from our current study and previous studies indicate that children were usually injured at home or at school^28–30^; patients aged 60 years or older usually had ocular trauma because of a fall at home^31^.

Open-globe injuries are those that involve penetration of the cornea/sclera and are more severe than closed-globe injuries^18^. In our patients hospitalized for major ocular trauma, the prevalence rates of open-globe injury were 68.0% and 73.6% in 2002–2004 and 2012–2014, respectively. By contrast, in studies of “all severities” of ocular trauma presenting to the emergency department, closed-globe injuries were more prevalent, with open-globe injuries reported to represent 6.3% of cases in northern Taiwan and 14.2% in Korea^19,32^; these findings can be attributed to the inclusion of patients with minor injuries, such as corneal abrasion or ocular surface foreign bodies, who were followed-up on an outpatient basis. Furthermore, open-globe injuries accounted for 41.6% of cases in Lee *et al*.’s national cohort study on hospitalized ocular trauma in Taiwan^6^, in which all levels of hospitals were included. Thus, the present study highlighted the significance of potentially vision-threatening ocular trauma treated at a university-affiliated tertiary referral medical centre. Another noticeable change in this study is that the proportion of hospitalized closed-globe injuries significantly decreased over a decade (30.2% and 18.6% in 2002–2004 and 2012–2014, respectively), a finding that we believe is largely attributable to the change in hospitalization criteria resulting in closed-globe injuries more often being treated on an outpatient basis.

In this study, anterior segment injuries were common and included corneal/scleral laceration or limbal wound dehiscence, hyphaemia, and lens injury, correlating with results reported in our previous study and other published studies^11,13,28,30,31,33^. On the other hand, posterior segment injuries, such as vitreous haemorrhage, retinal injury, and optic nerve injury, were seen less frequently in our study, but were associated with a worse final visual acuity^14,28^. The use of intracameral injection of antibiotics increased significantly in 2012–2014 compared to that in 2002–2004, and no patients developed endophthalmitis in the 2012–2014 period. In the past ten years, cefuroxime has been advocated for endophthalmitis prevention after cataract surgery. In a national prospective study in Sweden involving 225,471 cataract operations, the incidence of postoperative endophthalmitis was 0.045% in cases using intracameral cefuroxime, whereas the rate in patients who did not use cefuroxime was 0.350%^34^. In a multicentre prospective study performed by the European Society of Cataract and Refractive Surgeons (ESCRS), intracameral cefuroxime reduced the incidence of postoperative endophthalmitis by 4.92-fold in 16,603 cases of cataract surgery^35,36^. In a retrospective study of 46,292 cataract procedures, the odds ratio of the incidence of endophthalmitis for topical prophylaxis alone versus intracameral cefuroxime was 5.7^37^. We suggest that intracameral injection of cefuroxime can be considered in ocular trauma, particularly in anterior segment injuries.

The goals of emergency surgery to manage ocular trauma are to repair the wound and restore the anatomic structure of the eye. In some cases, a second or a series of procedures will be needed for maximal rehabilitation of vision or to treat complications or sequelae. Indications for subsequent surgeries include secondary implantation of an intraocular lens for traumatic/surgical aphakia, repair of retinal detachment, and penetrating keratoplasty to manage a corneal scar or bullous keratopathy. Subsequent procedures were performed in 15.4–18.2% of the patients in the two periods of this study, 9.6% of patients in Oum *et al*.’s study^19^, and 33% of patients in May *et al*.’s study^20^. We attribute these frequency differences to the differences in the type and severity of injuries in the study populations. The outcome of surgery for ocular trauma usually depends on the severity and extent of the initial injury, but careful evaluation and awareness of potential complications maximize the chances for ultimate surgical success.

In the two periods of this study, about half (46.8% and 47.8% in 2002–2004 and 2012–2014, respectively) of the patients had improved visual acuity after emergency treatment, whereas vision was unchanged in most remaining patients (48.7% and 43.0%, respectively). However, in a small proportion of patients (3.5% and 10.1%, respectively), the visual acuity was conversely worsened after the treatment. In our previous study and other published studies, the visual outcome after major ocular trauma was generally unsatisfactory^4,7,8,11–14,16,28,31^. Factors that have been documented to correlate with the visual prognosis after trauma include visual acuity immediately after the injury, presence of an afferent pupillary defect, type and mechanism of injury, location and extent of penetrating wounds, and presence of lens damage, vitreous haemorrhage, retinal detachment, intraocular foreign body, or endophthalmitis^14,28,38^. In addition, patients with multiple-site injuries are theoretically considered to have poorer visual outcomes than those with single-site injuries; however, this is strongly influenced by other more important factors such as the nature of the injury and the location and extent of the initial damage.

In the two periods of this study, the final visual acuity was poor (20/200 or worse) in more than one third (36.7% in 2002–2004 and 33.9% in 2012–2014) of patients. Even more, blindness (only light perception or no light perception) was observed in 11.8% and 15.7% out of all patients in 2002–2004 and 2012–2014, respectively. The visual outcome profile of our patients was similar to another report of hospitalized ocular trauma patients in Taiwan by Chang *et al*.^11^, but far less favourable than those in Tsai *et al*.’s report^32^ consisting of “all severities” of ocular trauma treated in the Emergency Department, in which 45.7% of patients had good final visual acuities of 20/40 or better, 24.9% had moderate visual acuities of 20/200 to 20/50, and 11.3% had poor visual acuities of 20/400 or worse. These results highlight the significant role that major ocular trauma plays in the aetiology of partial or complete vision loss. The high proportion of unsatisfactory outcomes after ocular trauma emphasizes the importance of education and other measures to prevent trauma.

Our study has some limitations. First, we included only patients hospitalized in the Department of Ophthalmology at a single medical centre with a primary diagnosis of major ocular trauma. The exclusion of those with injuries that were mild and treated on an outpatient basis unavoidably leads to an underestimation of the prevalence of ocular trauma among the population served by our hospital. Moreover, vehicle-related ocular trauma is theoretically more prevalent than the results of this study indicate, since some accident victims with ocular injuries or orbital injuries/fractures are admitted to the neurosurgery or plastic surgery department for care of more severe head or facial injuries and were hence not included in our subject population with a primary diagnosis of eye injury^6^. The second limitation relates to the study design. Information obtained by a retrospective review of medical records is inherently subject to inaccuracies or inconsistencies in terms of what data were obtained and in how they were interpreted or documented. Third, it is difficult to distinguish a true time trend versus inherent variability with only two three-year time spans of data. Future studies should assess at least three time spans of data to develop a trend-line.

## Conclusion

Ocular trauma remains an important cause of preventable visual morbidity and blindness. This study provides updated information on temporal changes in hospitalized major ocular injuries in a Taiwanese population over a decade. The individuals at the highest risk of eye injuries in both examined time periods were young and middle-aged males injured at work by a piece of metal striking the eye(s). The most common injuries remained anterior-segment open-globe injuries. Features that changed after a decade included average patient age, which increased by 10 years in the latter period, and the frequencies of fall injury, lacrimal system laceration, lens injury, corneal/scleral foreign bodies, and use of intracameral antibiotics during surgery. Of note, therapeutic advancements over the examined decade did not appear to improve visual outcomes, highlighting the importance of the nature and extent of the injury. This study contributes to the existing knowledge regarding the epidemiology of eye injuries, and can help clinicians better evaluate and manage ocular trauma, increase public awareness of risk activities and ocular protection, and help the government develop public health policies and education programs to prevent ocular trauma and improve the treatment thereof.
