# Author Correction: Major ocular trauma in Taiwan: 2002–2004 versus 2012–2014

**DOI:** 10.1038/s41598-018-31648-3

**Published:** 2018-09-05

**Authors:** Yi-Sheng Chang, Yu-Ti Teng, Yi-Hsun Huang, Mei-Ling Liu, Jia-Horung Hung, Sheng-Min Hsu, Fu-Chin Huang, Min-Hsiu Shih, Wan-Ju Chen, Chun-Chieh Lai, Shu-Fang Hsiao, Shih-Hao Wang, Sung-Huei Tseng

**Affiliations:** 1 Department of Ophthalmology, College of Medicine, National Cheng Kung University, Tainan, Taiwan; 2 Department of Ophthalmology, National Cheng Kung University Hospital, College of Medicine, National Cheng Kung University, Tainan, Taiwan; 3 Institute of Clinical Medicine, College of Medicine, National Cheng Kung University, Tainan, Taiwan

Correction to: *Scientific Reports* 10.1038/s41598-018-25030-6, published online 04 May 2018

The PDF and HTML versions of this Article contain extensive typographical errors in the reference citations in the Discussion section.

In the fourth paragraph,

“For the young-adult and middle-aged males in our study, activity risk factors for major ocular trauma were metal-working, driving, social behaviours such as fighting, and participation in dangerous sports or hobbies, similar to findings from previous reports^7,8,11–14,17,19^. In contrast, women in our study were most often injured at home and the workplace. A universal finding in studies of work-related eye injuries is that 55% to 91% of patients were not using protective eye wear at the time of injury^20–23^.”

should read:

“For the young-adult and middle-aged males in our study, activity risk factors for major ocular trauma were metal-working, driving, social behaviours such as fighting, and participation in dangerous sports or hobbies, similar to findings from previous reports^7,8,11–14,17,20^. In contrast, women in our study were most often injured at home and the workplace. A universal finding in studies of work-related eye injuries is that 55% to 91% of patients were not using protective eye wear at the time of injury^21–24^.”

Additionally,

“Most patients involved in traffic accidents are young adolescents riding motorcycles, a common occurrence in Taiwan^24^.”

should read:

“Most patients involved in traffic accidents are young adolescents riding motorcycles, a common occurrence in Taiwan^25^.”

In the fifth paragraph,

“By contrast, middle-age laborers engaging in high-risk work, such as construction, manufacturing, or agriculture, have elongated their working ages and are now retiring later than before^25^, thereby continuing to be exposed to high risks of ocular trauma over time. Second, a law implemented in 1997 enforcing motorcyclists to wear helmets has reduced the risk and severity of head and/or eye injuries decade by decade, particularly among young males^26,27^.”

should read:

“By contrast, middle-age laborers engaging in high-risk work, such as construction, manufacturing, or agriculture, have elongated their working ages and are now retiring later than before^26^, thereby continuing to be exposed to high risks of ocular trauma over time. Second, a law implemented in 1997 enforcing motorcyclists to wear helmets has reduced the risk and severity of head and/or eye injuries decade by decade, particularly among young males^27,28^.”

Additionally,

“The risk of ocular trauma related to working with hazardous materials or involvement in a traffic accident can be greatly reduced by such measures as using safety (laminated) glass in windscreens; wearing helmets, safety goggles, seat belts, and other protective gear appropriate to the high-risk activity^23^; and avoiding risky behaviours, such as drinking alcohol before driving or not following safety procedures for operating machinery. Education regarding these safety measures is essential to reduce the incidence and severity of ocular injuries. Evidence from our current study and previous studies indicate that children were usually injured at home or at school^28–30^; patients aged 60 years or older usually had ocular trauma because of a fall at home^31^.”

should read:

“The risk of ocular trauma related to working with hazardous materials or involvement in a traffic accident can be greatly reduced by such measures as using safety (laminated) glass in windscreens; wearing helmets, safety goggles, seat belts, and other protective gear appropriate to the high-risk activity^24^; and avoiding risky behaviours, such as drinking alcohol before driving or not following safety procedures for operating machinery. Education regarding these safety measures is essential to reduce the incidence and severity of ocular injuries. Evidence from our current study and previous studies indicate that children were usually injured at home or at school^29–31^; patients aged 60 years or older usually had ocular trauma because of a fall at home^32^.”

In the sixth paragraph,

“By contrast, in studies of “all severities” of ocular trauma presenting to the emergency department, closed-globe injuries were more prevalent, with open-globe injuries reported to represent 6.3% of cases in northern Taiwan and 14.2% in Korea^19,32^”

should read:

“By contrast, in studies of “all severities” of ocular trauma presenting to the emergency department, closed-globe injuries were more prevalent, with open-globe injuries reported to represent 6.3% of cases in northern Taiwan and 14.2% in Korea^19,20^”

In paragraph seven,

“In this study, anterior segment injuries were common and included corneal/scleral laceration or limbal wound dehiscence, hyphaemia, and lens injury, correlating with results reported in our previous study and other published studies^11,13,28,30,31,33^. On the other hand, posterior segment injuries, such as vitreous haemorrhage, retinal injury, and optic nerve injury, were seen less frequently in our study, but were associated with a worse final visual acuity^14,28^.”

should read:

“In this study, anterior segment injuries were common and included corneal/scleral laceration or limbal wound dehiscence, hyphaemia, and lens injury, correlating with results reported in our previous study and other published studies^11,13,29,31–33^. On the other hand, posterior segment injuries, such as vitreous haemorrhage, retinal injury, and optic nerve injury, were seen less frequently in our study, but were associated with a worse final visual acuity^14,29^.”

In the eighth paragraph,

“Subsequent procedures were performed in 15.4–18.2% of the patients in the two periods of this study, 9.6% of patients in Oum *et al*.’s study^19^, and 33% of patients in May *et al*.’s study^20^.”

should read:

“Subsequent procedures were performed in 15.4–18.2% of the patients in the two periods of this study, 9.6% of patients in Oum *et al*.’s study^20^, and 33% of patients in May *et al*.’s study^21^.”

In the ninth paragraph,

“In our previous study and other published studies, the visual outcome after major ocular trauma was generally unsatisfactory^4,7,8,11–14,16,28,31^. Factors that have been documented to correlate with the visual prognosis after trauma include visual acuity immediately after the injury, presence of an afferent pupillary defect, type and mechanism of injury, location and extent of penetrating wounds, and presence of lens damage, vitreous haemorrhage, retinal detachment, intraocular foreign body, or endophthalmitis^14,28,38^.”

should read:

“In our previous study and other published studies, the visual outcome after major ocular trauma was generally unsatisfactory^4,7,8,11–14,16,29,32^. Factors that have been documented to correlate with the visual prognosis after trauma include visual acuity immediately after the injury, presence of an afferent pupillary defect, type and mechanism of injury, location and extent of penetrating wounds, and presence of lens damage, vitreous haemorrhage, retinal detachment, intraocular foreign body, or endophthalmitis^14,29,38^.”

Finally, in the tenth paragraph,

“The visual outcome profile of our patients was similar to another report of hospitalized ocular trauma patients in Taiwan by Chang *et al*.^11^, but far less favourable than those in Tsai *et al*.’s report^32^ consisting of “all severities” of ocular trauma treated in the Emergency Department, in which 45.7% of patients had good final visual acuities of 20/40 or better, 24.9% had moderate visual acuities of 20/200 to 20/50, and 11.3% had poor visual acuities of 20/400 or worse.”

should read:

“The visual outcome profile of our patients was similar to another report of hospitalized ocular trauma patients in Taiwan by Chang *et al*.^11^, but far less favourable than those in Tsai *et al*.’s report^19^ consisting of “all severities” of ocular trauma treated in the Emergency Department, in which 45.7% of patients had good final visual acuities of 20/40 or better, 24.9% had moderate visual acuities of 20/200 to 20/50, and 11.3% had poor visual acuities of 20/400 or worse.”

